# Microwave Photonic Fiber Ring Resonator

**DOI:** 10.3390/s22103771

**Published:** 2022-05-16

**Authors:** Chen Zhu, Yiyang Zhuang

**Affiliations:** Research Center for Optical Fiber Sensing, Zhejiang Laboratory, Hangzhou 311100, China; yyzhuang@zhejianglab.com

**Keywords:** microwave photonics, optical fiber ring resonator, optical fiber sensor, strain sensor, bending sensor

## Abstract

In this article, a new concept of microwave photonic (MWP) fiber ring resonator is introduced. In particular, the complex transmission spectra of the resonator in the microwave domain, including magnitude and phase spectra, are measured and characterized. Multiple resonance peaks are obtained in the magnitude spectrum; rapid variations in phase near resonance (i.e., enhanced group delay) are observed in the phase spectrum. We also experimentally demonstrate that the MWP fiber ring resonator can be potentially employed as a novel optical fiber sensor for macro-bending and fiber length change sensing (strain sensing). The experimental results are in good agreement with theoretical predictions.

## 1. Introduction

Optical ring resonators are critical optical devices and have found many important applications [1,2,3,4]. In recent years, optical-fiber-based ring resonators have attracted great interest and have been widely used in both passive and active optical devices, such as lasers [5], filters [6], and sensors [7,8,9,10]. Compared to on-chip micro-ring resonators, optical-fiber-based ring resonators are usually larger in size. However, optical-fiber ring resonators can be readily constructed using off-the-shelf optical-fiber components, e.g., fiber couplers, allowing systematic investigation of their optical characteristics [11,12]. A fiber ring resonator is typically characterized by measuring its intensity transfer function, which is accomplished by probing the resonator using a highly coherent light source. Importantly, the state of polarization of the probing light must be tightly controlled. Meanwhile, the wavelength of the probing light must be varied in a certain range to acquire the entire coherent transfer function at resonance. Additionally, the linewidth of the light source must be at least one order of magnitude smaller than the resonance width of the resonator, such that the resonance can be resolved. The requirement on the linewidth of the light source becomes a seriously limiting factor when it comes to a fiber ring resonator with a long cavity length [13]. To relieve the stringent requirement on the light source, optical time-domain reflectometry (OTDR) was reported as a complementary technique for the characterization of fiber ring resonators [13].

Microwave photonics (MWP), an emerging interdisciplinary subject, has undergone tremendous growth and advancements over the past two decades [14,15,16], and has found successful applications in signal generation [17], signal processing [18], as well as sensing [19,20]. Of particular relevance here, various fiber optic sensors based on different MWP interrogation methods were developed recently, such as MWP-filtering techniques [21,22,23], optical-carrier-based microwave interferometry [16,24], optoelectronic oscillators [25,26], phase-shift amplified interferometry [27,28], etc. In this paper, we propose and demonstrate MWP fiber ring resonators. Discrete resonance peaks can be observed in the transmission spectrum in the microwave domain of an MWP fiber ring resonator. Similar to optical-domain ring resonators, rapid variations in phase near resonance are also observed in the MWP fiber ring resonators, making them a potential platform for slow–fast light investigation. Additionally, the capability of the MWP fiber ring resonator for potential sensing applications is also demonstrated.

## 2. Principle of System

**Figure 1:**
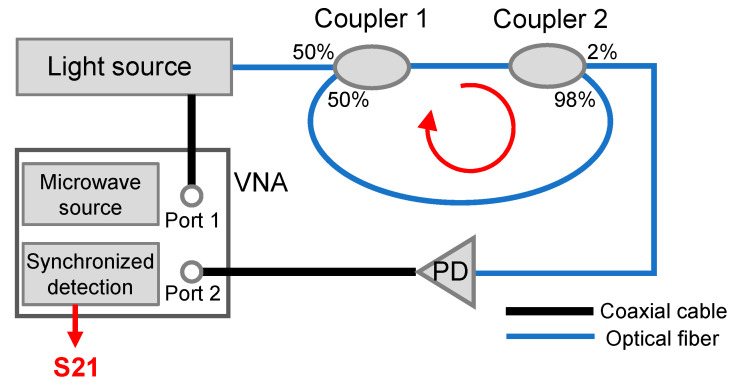
Schematic diagram of the proposed MWP fiber ring resonator. VNA, vector network analyzer; PD, photodetector.

Figure 1 shows a schematic diagram of the MWP fiber ring resonator based on a direction–modulation system. The optical output from the light source is intensity-modulated by the microwave signal from port 1 of a vector network analyzer (VNA). The intensity-modulated light is sent into a 1 × 2 optical fiber coupler, i.e., coupler 1, with a coupling ratio of 50/50. Another fiber coupler (coupler 2) with a coupling ratio of 98/2 is connected to coupler 1. The output from 2% branch of coupler 2 is directed to a photodetector (PD), and the electrical signal is then sent to port 2 of the VNA. The 98% branch of coupler 2 is connected to coupler 1, where an optical fiber ring is formed. After sweeping the microwave modulation frequency and recording the corresponding magnitude and phase information at each of the discrete frequencies, the complex transmission spectrum of the fiber ring resonator in the microwave domain can be obtained, i.e., S21 from the VNA.

Note that essentially only a single optical-fiber coupler is required to form a fiber ring. However, in this work, two couplers were utilized because it is more convenient to adjust the coupling coefficients of the fiber ring resonator, as illustrated later. A complete mathematical model for the proposed MWP fiber ring resonator was developed as follows.

Assume the electric field of the intensity-modulated optical signal is given by

(1) $$E_{0}=\sqrt{1+M\cos\left(2\pi ft\right)}\cdot A\exp\left(-j\omega t\right)$$

where *M* and *A* are the amplitudes of the microwave modulation signal and the optical carrier, respectively; *f* is the frequency of the microwave signal; *ω* is the angular frequency of the optical carrier; and *t* is the time term. After each trip of the optical signal in the fiber ring, a small portion of the signal (e.g., 2%) is coupled out of the fiber ring and sent for detection. The total power of the transmitted optical signal can be expressed as

(2) $${\left|E_{total}\right|}^{2}=\frac{1}{\Delta \omega }\left({\int_{\omega_{\min}}^{\omega_{\max}}\left|\sum_{i=1}^{N}E_{i}\right|}^{2}d\omega +\int_{\omega_{\min}}^{\omega_{\max}}\sum_{i=1,j=1,i\neq j}^{N}\left(E_{i}E_{j}{}^{*}+E_{i}{}^{*}E_{j}\right)d\omega \right)$$

where *N* represents the total number of trips of the optical signal in the fiber ring; Δω is the frequency bandwidth of the light source and is equal to *ω*_max_ − *ω*_min_; and *E_i_* is given by

(3) $$\begin{array}{c} E_{i}=\tau^{i}\sqrt{1+M\cos\left\{2\pi f\left[t+\frac{nL_{0}+(i-1)nL}{c}\right]\right\}}\\ \cdot A\exp\left\{-j\omega \left[t+\frac{nL_{0}+(i-1)nL}{c}\right]\right\} \end{array}$$

where *L*_0_ is the length of the fiber line connecting coupler 1 and coupler 2; *n* and *L* are the refractive index of the optical fiber and total length of the fiber ring; and *τ* denotes the amplitude transmission factor of the fiber ring. Importantly, in the MWP system, the length of the fiber ring (e.g., a few meters) is set to be sufficiently larger than the coherence length of the light source (on the order of mm) so that the cross-product term in Equation (2) approaches zero [29]. On the other hand, the self-product term in Equation (2) can be written as

(4) $$\begin{array}{l} \frac{1}{\Delta \omega }{\int_{\omega_{\min}}^{\omega_{\max}}\left|\sum_{i=1}^{N}E_{i}\right|}^{2}d\omega =\sum_{i=1}^{N}\tau^{2i}A^{2}\\ \;+\sum_{i=1}^{N}\tau^{2i}A^{2}M\cos\left[2\pi f(t+\frac{nL_{0}+(i-1)nL}{c})\right] \end{array}$$

After optoelectronic conversion (with a coefficient of *g*) and synchronized detection, the total can be expressed as

(5) $$S=A_{eff}\cos(2\pi ft+\phi_{eff})$$

where

(6) $$\begin{array}{l} A_{eff}=g\sqrt{\sum_{i=1}^{N}\sum_{j=1}^{N}\tau^{2i}\tau^{2j}M^{2}A^{4}\cos\left[2\pi f\left(i-j\right)\frac{nL}{c}\right]}\\ \phi_{eff}=\tan^{-1}\frac{\sum_{i=1}^{N}\tau^{2i}\sin\left[2\pi f\frac{nL_{0}+(i-1)nL}{c}\right]}{\sum_{i=1}^{N}\tau^{2i}\cos\left[2\pi f\frac{nL_{0}+(i-1)nL}{c}\right]} \end{array}$$

The magnitude spectrum *A_eff_* essentially gives the transmission spectrum of the fiber ring resonator in the microwave domain. According to Equation (6), the phase-matching condition of the fiber ring resonator is given by

(7) $$2\pi f\frac{nL}{c}=2k\pi ,\;k=1,2,3\ldots$$

Therefore, the free spectral range (*FSR*) of the MWP fiber ring resonator can be expressed as

(8) $$FSR=\frac{c}{nL}$$

which is the same as conventional optical ring resonators. It is worth mentioning that the phase spectrum can also be measured using the VNA, from which the group delay of the signal can be obtained.

## 3. Results and Discussion

### 3.1. System Implementation and Characterization

**Figure 2:**
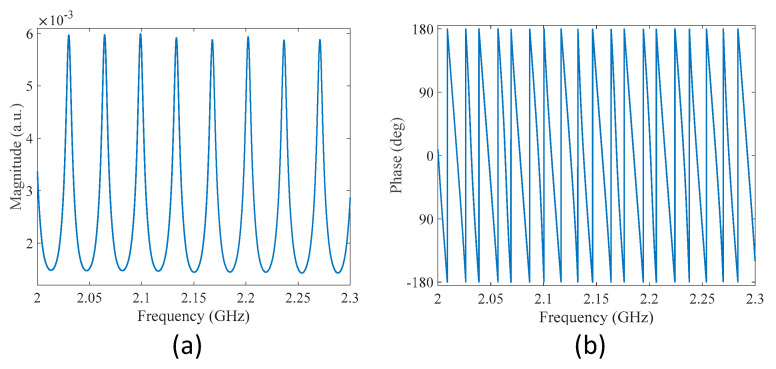
Measured transmission spectra of the MWP fiber ring resonator. (**a**) Magnitude spectrum. (**b**) Phase spectrum.

The system illustrated in Figure 1 was implemented using a direct modulation laser, a PD, and a portable VNA. The total length of the fiber ring *L* was set to approximately 6 m. All the measurements were performed at room temperature (20 ± 0.5 °C). Figure 2 gives the measured transmission spectra of the MWP fiber ring resonator in the frequency range 2–2.3 GHz, including magnitude spectrum and phase spectrum given in Figure 2a,b, respectively. Multiple peaks could be observed from the magnitude spectrum, corresponding to the resonance frequencies of the fiber ring resonator. The *FSR* was found to be 34.4 MHz, which was close to the predicted value of 34.1 MHz based on Equation (8), given the refractive index and length of the fiber ring to be 1.468 and 6 m, respectively. The fringe visibility of the transmission spectrum was approximately 12 dB. The quality factor (Q-factor) of the resonance peak at 2.03 GHz and the finesse of the fiber ring resonator were determined to be approximately 287 and 5, respectively. The relatively low fringe contrast is due to the two unbalanced couplers used in the fiber ring; the small Q-factor is a result of the high transmission loss caused by the 50/50 coupler. The two measures of the fiber ring resonator (i.e., finesse and Q-factor) can be further improved by replacing coupler 1 with another coupler with a coupling ratio of 98/2.

**Figure 3:**
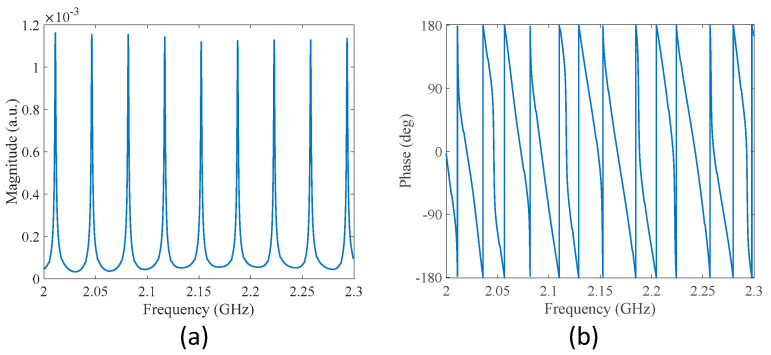
Measured transmission spectra of the modified MWP fiber ring resonator. (**a**) Magnitude spectrum. (**b**) Phase spectrum.

Figure 3 shows the measured transmission spectra of the modified MWP fiber ring resonator, where coupler 1 was replaced using a coupler with a coupling ratio of 98/2. The 2% branch was connected to the light source, and the 98% branch was connected to coupler 2. The total length of the fiber ring was reduced by 0.2 m during the fiber-splicing process, resulting in a total length of 5.8 m, corresponding to a reduction of 3.3% in fiber ring length. The *FSR* of the magnitude spectrum was found to be 35.3 MHz, as can be expected from Equation (8). As can be seen from Figure 3a, the Q-factor and the finesse of the modified fiber ring resonator were both increased compared to the previous one, and were found to be approximately 1260 and 22, respectively. It is also interesting to see that more features can be observed in the phase spectrum shown in Figure 3b, as discussed later.

**Figure 4:**
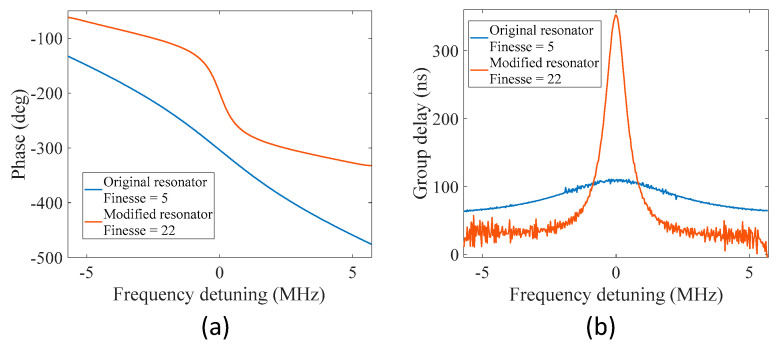
Phase shift and group delay near resonance of the MWP fiber ring resonator. (**a**) Phase variations. (**b**) Group delay.

In optical ring resonators, the phase of the optical signal undergoes a rapid variation near each of the resonance frequencies of the ring resonator. For the proposed MWP fiber ring resonator, rapid variations in the phase were also observed near each resonance in the microwave domain. Figure 4a gives the enlarged view of the unwrapped phase spectra shown in Figure 2b and Figure 3b centered at one of its normalized resonance frequencies. The calculated group delay as a function of frequency is shown in Figure 4b. For the modified fiber ring resonator with a larger finesse of 22, faster variations in phase were observed near the resonance frequency, resulting in a larger group delay at the resonance. Meanwhile, there is a tradeoff between the phase delay and the bandwidth of the curve, as is the case for optical ring resonators. It is interesting to see that by modulating the microwave signal onto an optical carrier, it is possible to manipulate the group delay (and velocity) of the microwave signal by sending the intensity-modulated optical signal to an MWP fiber ring resonator.

### 3.2. Bending Measurement

**Figure 5:**
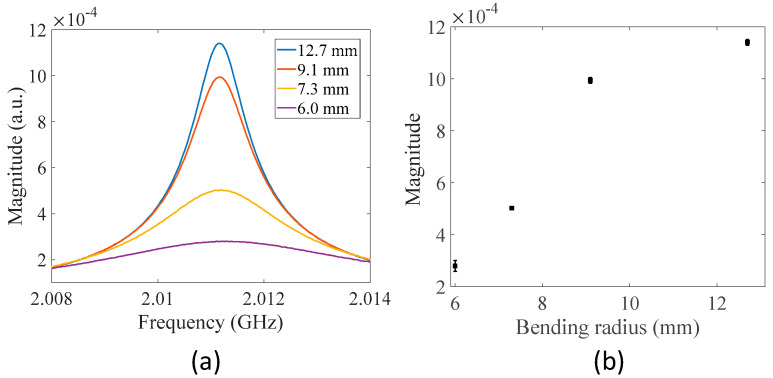
Responses of the MWP fiber ring resonator to fiber bending in the fiber ring. (**a**) Measured transmission spectra for different settings of bending radius of a small fiber section in the fiber ring. (**b**) Transmission magnitude at resonance with respect to bending radius. Each error bar indicates the maximum deviation of four independent measurements for each setting of bending radius.

As can be observed from Equation (5), the transmission spectrum of the MWP fiber ring resonator depends on the amplitude transmission factor of the fiber ring. To demonstrate the response, a small section of the fiber ring of the modified MWP resonator was bent to different diameters and the corresponding transmission spectra were measured. Figure 5a shows the recorded transmission spectra of the resonator in the magnitude for different bending radii. As the bending radius decreased, additional transmission loss was introduced to the fiber ring, decreasing the amplitude transmission factor. Therefore, the magnitude at resonance decreased with decreasing bending radii. Figure 5b plots the transmission magnitude at resonance with respect to bending radius. The results verify that the MWP fiber ring resonator responds to additional transmission loss introduced in the fiber ring induced by external perturbations (i.e., bending in this case), which also indicates that the MWP fiber ring resonator can be potentially used as a macro-bending sensor after proper calibration.

### 3.3. Length Change Measurement

**Figure 6:**
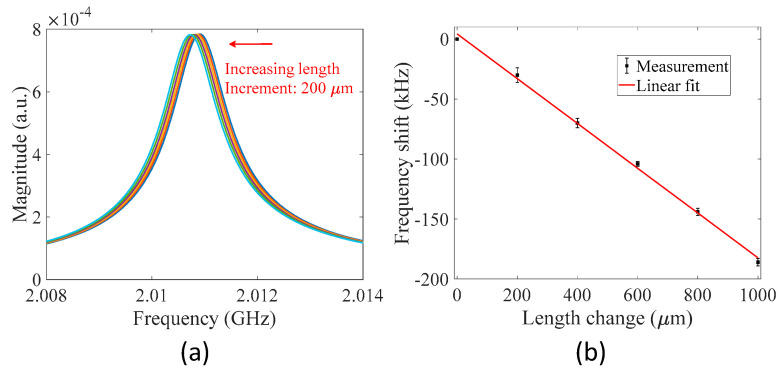
Responses of the MWP fiber ring resonator to length change in the fiber ring. (**a**) Measured transmission spectra for different settings of length change. (**b**) Shift in resonance frequency as a function of length change. Each error bar indicates the maximum deviation of four independent measurements for each setting of length change. The measurement uncertainty is mainly due to the system noise and uncertainty introduced in the data processing (determination of resonance frequency) as well as the environmental perturbations (e.g., temperature fluctuations and experimental setup vibrations) and experimental errors.

Next, an experiment was performed to demonstrate that the MWP fiber ring resonator also responds to length changes in the fiber ring. An MWP fiber ring resonator with a fiber ring length of ~5.8 m was constructed in the experiment. Two points of the optical fiber in the fiber ring were fixed to two translation stages with a distance of 300 mm. Stress was applied to the secured optical fiber section to elongate the fiber in steps of 200 µm. A total length change of 1000 µm was applied in the experiment, corresponding to a relative length change of 1.72 × 10^−4^, provided the initial length of the fiber was 5.8 m. Figure 6a gives the measured transmission spectra of the resonator centered at the resonance frequency of ~2.01 GHz for different settings of length change. The transmission spectrum shifted to the low-frequency region with increasing length of the fiber ring, as can be expected from Equation (7). The shift in resonance frequency as a function of length change is plotted in Figure 6b. A linear curve fit was applied to the measured data points, and the slope of the fitted curve was determined to be −0.1866 kHz/µm with R-squared of 0.9977. The results demonstrate that the MWP fiber ring resonator can be used as a strain sensor with flexible gauge length (e.g., from cm scale to hundreds of meters) after proper calibration. Note that the MWP fiber ring resonator is also sensitive to changes in temperature as temperature variations vary the optical length of the fiber ring due to the thermo-optic effect and thermal expansion effect. Thus, in real-world applications, temperature compensation is required.

## 4. Conclusions

To conclude, we have proposed and experimentally demonstrated the concept of the MWP fiber ring resonator. Discrete resonance peaks were observed in the magnitude transmission spectrum of the MWP fiber ring resonator in the microwave domain. Rapid variations in phase near resonance were also verified in the phase spectrum of the resonator, similar to conventional optical resonators. Responses of the MWP ring resonator to variations in the bending radius of the fiber and length change of the fiber ring were experimentally investigated, verifying the potential of the MWP resonator as a novel optical fiber sensor for macro-bending and strain measurements.

## Data Availability

Not applicable.
